# Differential expression of Mad2 gene is consequential to the patterns of histone H3 post-translational modifications in its promoter region in human esophageal cancer samples

**DOI:** 10.18632/oncotarget.28554

**Published:** 2024-02-05

**Authors:** Chongtham Sovachandra Singh, Nabamita Boruah, Atanu Banerjee, Sillarine Kurkalang, Pooja Swargiary, Hughbert Dakhar, Anupam Chatterjee

**Affiliations:** ^1^School of Biosciences, The Assam Royal Global University, Guwahati 781035, India; ^2^Perelman School of Medicine, University of Pennsylvania, Philadelphia, PA 19104, USA; ^3^Department of Zoology, LN Mithila University, Darbhanga, Bihar 846008, India; ^4^Comprehensive Cancer Center, University of Chicago Medicine, Chicago, IL 60637, USA; ^5^Histopathology Division, Nazareth Hospital, Laitumkhrah, Shillong 793003, India; ^6^Department of Biotechnology and Bioinformatics, Molecular Genetics Laboratory, North-Eastern Hill University, Shillong 793022, India

**Keywords:** Mad2 gene, histone methylation, histone acetylation, Rb-phosphorylation, esophageal cancer

## Abstract

Raw areca nut (AN) consumption increases esophageal squamous cell carcinoma (ESCC) due to overexpression of securin (pituitary tumor transforming gene1), causing chromosomal instability. Mitotic arrest deficient protein 2 (Mad2), a crucial spindle assembly checkpoint protein, is at risk of aneuploidy and tumor development when overexpressed or underexpressed. This study evaluates Mad2 status in human ESCC with AN consumption habits, revealing unclear molecular mechanisms. Human ESCC samples (*n* = 99) were used for loss of heterozygosity analysis at 4q25-28, while 32 samples were used for expression analysis of Mad2, E2F1 genes, and Rb-phosphorylation. Blood samples were used for metaphase preparation. The Mad2 deregulation was assessed using chromatin immunoprecipitation-qPCR assay in the core promoter region, establishing its association with the pRb-E2F1 circuit for the first time. The study revealed overexpression and underexpression of Mad2, premature anaphase, and chromosome missegregation in all the samples. LOH pattern identified a deletion in D4S2975 in 40% of ESCC samples. The study reveals the deregulation of pRb-E2F1 circuit in all samples. 4q27 disruption could be a factor for Mad2 underexpression in AN-induced esophageal carcinogenesis, while overexpression may be due to the deregulation of the Rb-E2F1 circuit and consequently elevation of H3K4me3 and H3K9ac. Mad2 expression levels with chromosomal abnormalities can be a clinical biomarker, but further research is needed to understand pRb’s role in Mad2 down-regulation.

## INTRODUCTION

Impaired spindle assembly checkpoints (SAC) lead to abnormal mitosis, causing aneuploid chromosomes and chromosome instability, a hallmark of human cancers [1, 2]. Mitotic arrest deficient protein 2 (Mad2) is one of the important SAC proteins, that inhibits anaphase-promoting complex by sequestering Cdc20 when chromosomes fail to attach mitotic spindle [3]. This way, Mad2 delays the anaphase onset and ensures proper chromosomal segregation [4]. It has been demonstrated that overexpression of Mad2 is associated with aneuploidy and tumorigenesis and is reported in many human cancers [5, 6]. On the other hand, Mad2 inactivation increases chromosome loss, promoting tumor formation and evolution [7]. Therefore, both either increased or decreased SAC gene expression, including Mad2, induce aneuploidy and tumor development in humans and mice [5, 8].

In India, people of the northeastern region, consume betel quid consisting of raw areca nut (AN) with a slaked lime wrapped in a betel leaf without tobacco. This unprocessed raw AN consists of higher alkaloids, polyphenols, and tannins compared with the dried one [9]. People often swallow the entire betel-quid after chewing, which is believed to contribute to the development of oral, esophageal, and gastric cancers. Increased consumption induces precocious anaphase (premature separation of sister chromatids), chromosomal instability, p53 and securin upregulation, and has been linked to oral, esophageal, and gastric cancers [10–13]. Therefore, these parameters can serve as a screening marker for identifying mitotic checkpoint defects in the early stages of AN exposure. Securin, a multifunctional protein, can be used as screening marker for mitotic checkpoint defects in early AN exposure, as overexpression has been linked to various cancers [14]. Securin overexpression is also linked to poor overall survival in malignant tumors like esophageal, hepatocellular carcinoma, and gastric cancer [15–17]. Recent epigenetic studies show elevated histone H3K4 trimethylation and acetylation at the H3K9 and H3K18 residues globally indicating increased transcriptional activation in the stomach tissue of mice after AN and lime exposure [13].

This study aims to assess the status of Mad2 in human esophageal cancer samples, as the molecular mechanism behind Mad2 deregulation remains unclear. Therefore, the present study investigates the relationship between Mad2 deregulation and the Rb-E2F1 circuit and epigenetic histone modification patterns in the Mad2 gene promoter region in human esophageal cancer samples. Previous research aimed to understand Mad2 deregulation during tumorigenesis, attributed to genomic rearrangements, altered gene dosage, promoter methylation, and transcriptional and translational defects [18, 19].

Interestingly, Mad2 gene mutations are rare in cancer [20] and therefore Mad2 expression with proper control is crucial for normal growth. Thus, there is an unmet need to systematically evaluate the Mad2 expression status and its relationship with cancer prognosis in the esophageal cancer samples from raw AN chewers where Securin upregulation was observed during the early days of AN exposure [11, 12]. For the first time, the aberrant expression status of the Mad2 gene was evaluated by assessing posttranslational histone H3 modifications by chromatin immunoprecipitation-qPCR (ChIP-qPCR) in the promoter region of the Mad2 gene and establishing its association with pRb-phosphorylation, and E2F1 expression in esophageal cancer tissues from patients with raw AN consumption habit. The present data reveal both over- and under-expression of Mad2 in the cancer tissues, and all showed premature anaphase and chromosome missegregation. Interestingly, the histone H3 modification pattern in the promoter region of Mad2 was in line with the Mad2 expression patterns, however, increased Rb inactivation and E2F1 release were observed in all the samples, irrespective of the Mad2 expression status.

## RESULTS

### General observations

The study utilized 131 esophageal cancer biopsies from two groups at different time points, and primarily these samples are well-differentiated squamous cell carcinoma.

### Deregulation of Mad2 expression analysis

**Figure 1:**
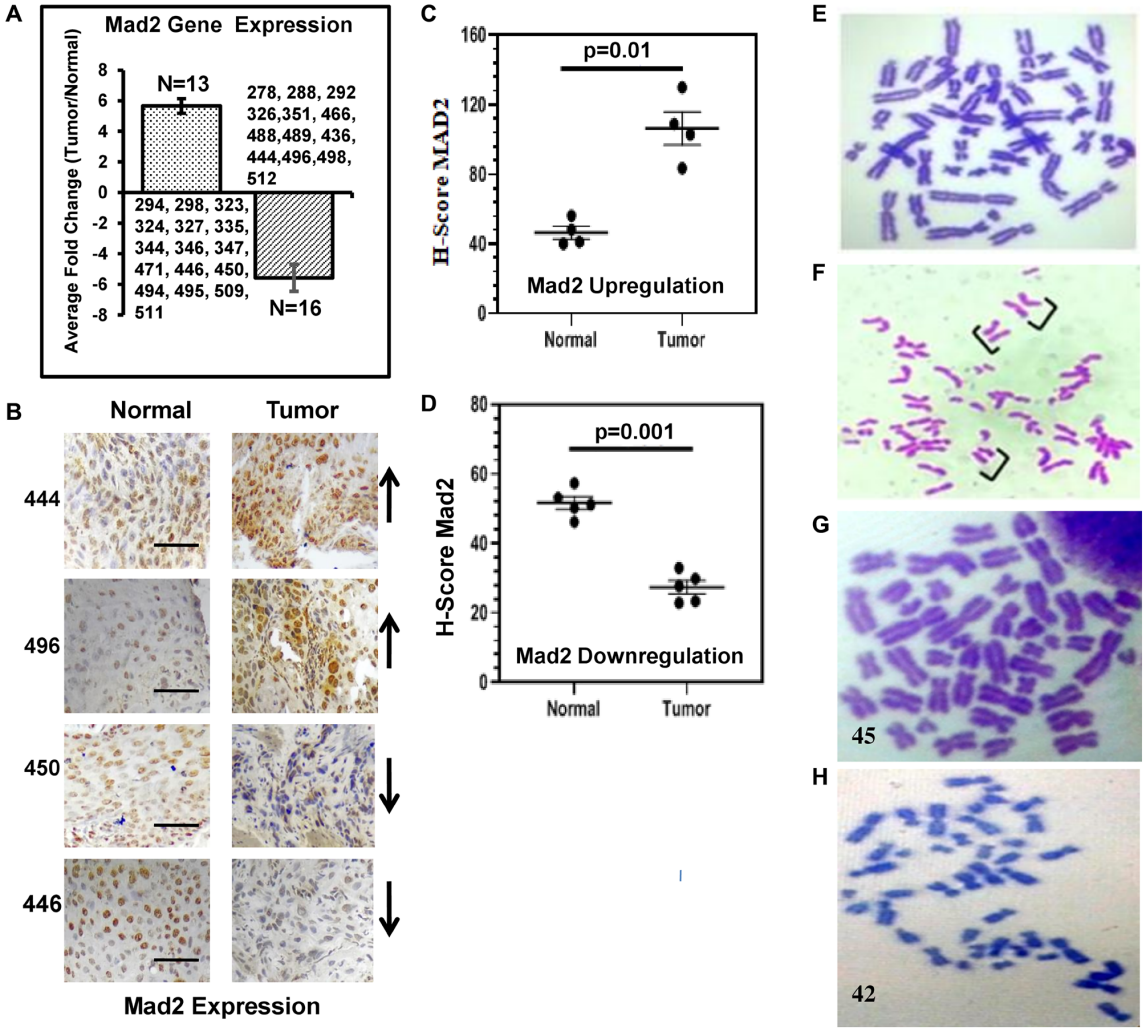
Expression analysis of the human Mad2 gene and karyotype analysis of genomic instability in esophageal cancer patients. (**A**) Expression of Mad2 gene in esophageal cancer tissues analyzed by qRT-PCR. Patients ID numbers are shown. (**B**) Representative images of an immunohistochemical (IHC) analysis of tumour and adjacent normal tissues in ESCC done with anti-Mad2 antibody. Patients ID numbers are shown in the left side. Arrows indicate the upregulation and down regulation of Mad2 expression in the tumor tissues. The magnification of all these images is 40x. (**C**, **D**) Scatterplot of H-scores based on IHC for Mad2 positive cells in Mad2 upregulation and Mad2 downregulation groups, respectively. (E) Normal metaphase spread from human PBLs. Data were analyzed using one-way ANOVA with Tukey’s multiple comparison post-tests. *P* values less than 0.05 are considered significant. The scale bar: 200 μm. (**F**) Premature sister-chromatid separation in PBL of esophageal cancer patients. Brackets show sister chromatids lying separated in mitotic figures that show the phenotype. (**G**, **H**) Metaphase spread showed 45 and 42 chromosomes in PBLs of esophageal cancer patients, respectively.

A total of 29 samples out of 32 cancer biopsies showed deregulation of the expression of Mad2 gene (Figure 1A). Significant overexpression of the Mad2 gene was found in 13 samples, whereas 16 samples showed significant downregulation. Three samples did not show any significant change in Mad2 expression between normal and tumor tissues.

Nine samples of 29 samples were used for the immunohistochemical (IHC) staining study to validate the qRT-PCR observation with respect to Mad2 gene expression. Four samples were selected from the Mad2 overexpression group and five were selected from the downregulated group. The H-score in normal tissue was around 45, while in tumor tissues in the Mad2 overexpression group, it reached 108. In the downregulated group, the H-score of the normal tissue was 55, which was reduced to 25 in tumor tissues (Figure 1B–1D).

### Studies on metaphase spreads

The study examined the impact of raw AN and lime consumption on chromosomes in esophageal cancer patients, finding that approximately 9% of mitotic figures had precocious anaphase in peripheral blood lymphocytes (PBL) of esophageal cancer patients fixed at 88 h irrespective of Mad2 expression status (Table 1). Such mitotic figures were absent in blood lymphocytes from ten non-chewers. However, precocious anaphase frequency varied inter-individually without age association of the individual. Chromosome counts showed stable karyotypes in non-chewers.

**Table 1:** Chromosome analysis of human PBLs from esophageal cancer patients with over- and under-expressed Mad2 gene

Habit (# subjects)	Mean Age (years) ± SD	Mad2 expression	Total metaphases scored ± SD	Premature anaphase separation (%) ± SEM	Aneuploidy % ± SEM
Non RAN Chewers (10)	47 ± 15	Normal	119 ± 14	0.1	0
RAN Chewers Only (04)	49 ± 05	Over expressed	123 ± 15	7.7 ± 0.3^*^	5.5 ± 0.4^*^
RAN+Tobacco (09)	50 ± 19	Over expressed	124 ± 10	8.3 ± 0.6^*^	6.8 ± 0.6^*^
RAN Chewers Only (06)	55 ± 12	Under expressed	116 ± 9	8.2 ± 0.6^*^	5.7 ± 0.7^*^
RAN+Tobacco (10)	60 ± 08	Under expressed	111 ± 8	9.8 ± 0.5^*^	8.8 ± 0.5^*^
RAN+Tobacco (03)	52 ± 08	NO significant Change	110 ± 8	8.0 ± 0.6^*^	6.3 ± 0.9^*^

^*^statistically significant in unpaired *t*-test.

Approximately, 9% of precocious anaphase and 8% of aneuploid cells were observed in the PBL of esophageal cancer patients with both Mad2 over- and under-expression (Figure 1F–1H). The present data demonstrated a significant induction of precocious anaphases and aneuploidy in PBL of esophageal cancer patients, irrespective of the expression status of the Mad2 gene. Interestingly, 3 cancer samples showed similar levels of precocious anaphases and aneuploid cells without showing any change in Mad2 expression compared to normal cells.

### pRb, E2F1 and Securin expression analysis through immunohistochemical staining

**Figure 2:**
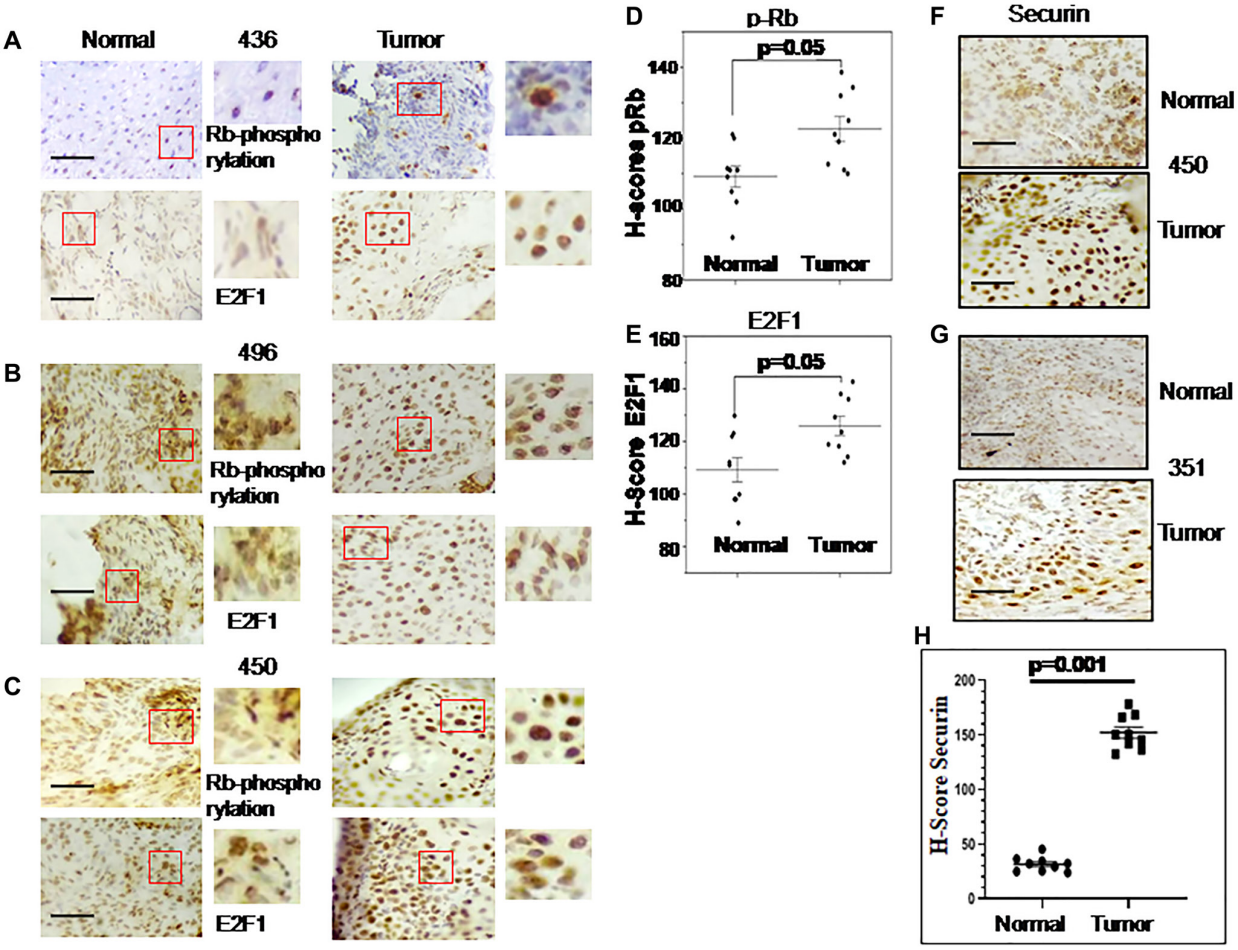
Immunohistochemical (IHC) images for Rb phosphorylation, E2F1 and securin gene expression in esophageal cancer and adjacent normal tissues. Representative images of an IHC analysis done with anti-Rb-phosphorylation and anti-E2F1 antibodies are shown in (**A**–**C**). Patients ID numbers are shown in the middle. (**D**) Rb-phosphorylation and (**E**) E2F1 expression levels in tumors and adjacent normal tissues analysed by H-score are shown as scatterplot. (**F**, **G**) show IHC images of Securin expression in the tumor and adjacent normal tissues. Patients ID numbers are shown on the right side. (**H**) Scatterplot shows the expression levels of Securin analysed by H-score. The magnification of all these images is 40x and for the marked area is 100x. Grouped scatterplot illustrating quantitative values within each grouping of H-scores which are represented as the mean ± SEM; *P*-values were calculated with untreated control using one-way ANOVA with Tukey’s multiple comparison pos*t*-tests. *P* values less than 0.05 are considered significant. The scale bar: 200 μm.

Immunohistochemical analysis is illustrated in Figure 2A–2E for the expression of pRb and E2F1 in normal and esophageal tumor cells. H-score indicates the significant increase of Rb-phosphorylation and consecutive stimulation of E2F1 expression in all the cancer samples compared to their normal counterpart.

Securin expression in ESCC (*n* = 9) patient samples was studied by immunostaining and found to be significantly higher in esophageal tumor sections compared to adjacent normal samples (Figure 2F, 2G). The H-score of Securin varied between 22 to 48 for normal samples and 136 to 186 for esophageal cancer samples (Figure 2H).

### Loss of heterozygosity analysis of 4q25-28

**Figure 3:**
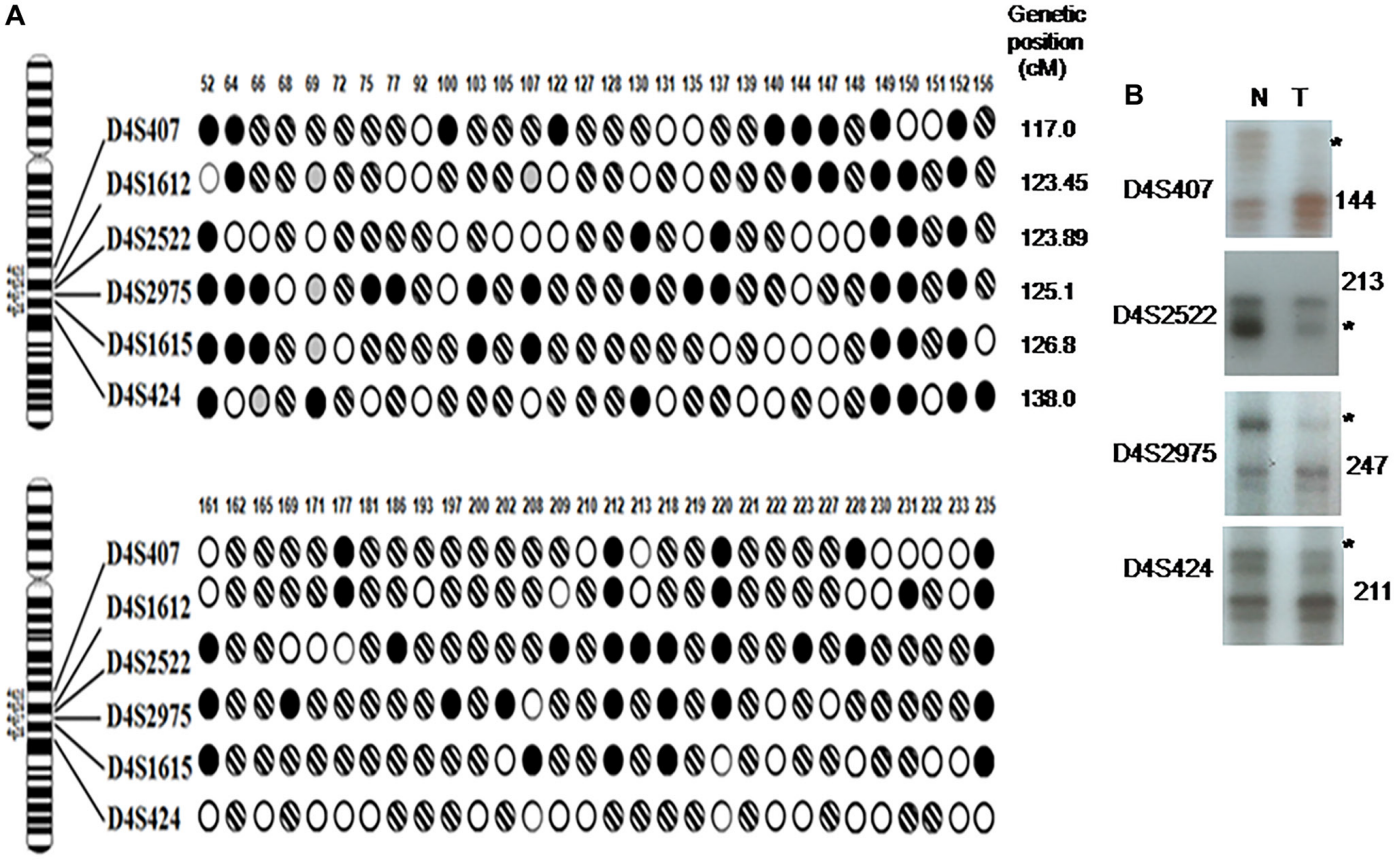
LOH on 4q25-28 in esophageal cancer samples. (**A**) G-banded ideogram is shown on the left, and the corresponding polymorphic loci are shown to the right of the ideogram. Patterns of deletions are indicated by circles below the sample numbers corresponding to each marker: ●, LOH; hatched circle, retention of heterozygosity ○; Empty circle, homozygous and uninformative; 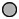, not done. Genetic position of each microsatellite marker is given on the right side. (**B**) Representative gels showing loss of heterozygosity in raw AN-associated (with and without tobacco) esophageal carcinoma in 4q25-28 region. N-normal blood DNA; T-tumor DNA; ^*^-indicating LOH. Tumor numbers and markers are shown in left.

Evaluation of LOH on a panel of 99 esophageal cancer tissues with 6 STRP markers mapped to chromosome 4q found deletions in at least one marker in 41 (62%) patients with the habit of AN and tobacco users and 51% in the tumors of only AN chewers (Table 2). Of the 57 tumors that had LOH on 4q, 12 (21%) showed LOH at all of the informative markers, suggesting 4q partial monosomy. The pattern of LOH in 4q25-28 region of 60 samples is shown in Figure 3A. Figure 3B shows LOH in the representative gels. The LOH pattern identified a maximum deletion at 4q27 ranging from D4S2975 to D4S1615 locus, with no association with patients’ age or sex. Loss in microsatellite markers D4S2975 at 4q27, which is located close to the Mad2 gene was noted in approximately 40% of ESCC samples.

**Table 2:** Frequency of LOH on chromosome 4q in esophageal carcinoma

Habit	Chromosome band	Locus	Genetic position cM	Heterozygosity (%)	# Studied/noninformative	LOH (%)
RAN+Tobacco	4q25	D4S407	117.0	87	66/12	15 (27.8)
	4q26	D4S1612	123.45	72	64/14	12 (24.0)
	4q26	D4S1522	123.89	59	66/16	16 (32.0)
	4q27	D4S2975	125.1	83	65/08	23 (40.4)
	4q27	D4S1615	126.8	75	65/13	15 (29.4)
	4q28	D4S424	138.0	83	65/26	08 (20.5)
RAN Only	4q25	D4S407	117.0	87	32/05	08 (29.6)
	4q26	D4S1612	123.45	72	31/03	06 (20.7)
	4q26	D4S1522	123.89	59	32/09	06 (26.1)
	4q27	D4S2975	125.1	83	33/07	10 (37.0)
	4q27	D4S1615	126.8	75	33/03	07 (27.0)
	4q28	D4S424	138.0	83	33/14	05 (26.3)

Abbreviations: cM: centiMorgan; LOH: loss of heterozygosity.

### ChIP analysis of chromatin composition

ChIP assay was conducted on esophageal tumor tissues and adjacent normal tissues to assess the recruitment of posttranslational modifications of histone H3 (methylation/acetylation of histone H3) in the securin gene promoter region.

**Figure 4:**
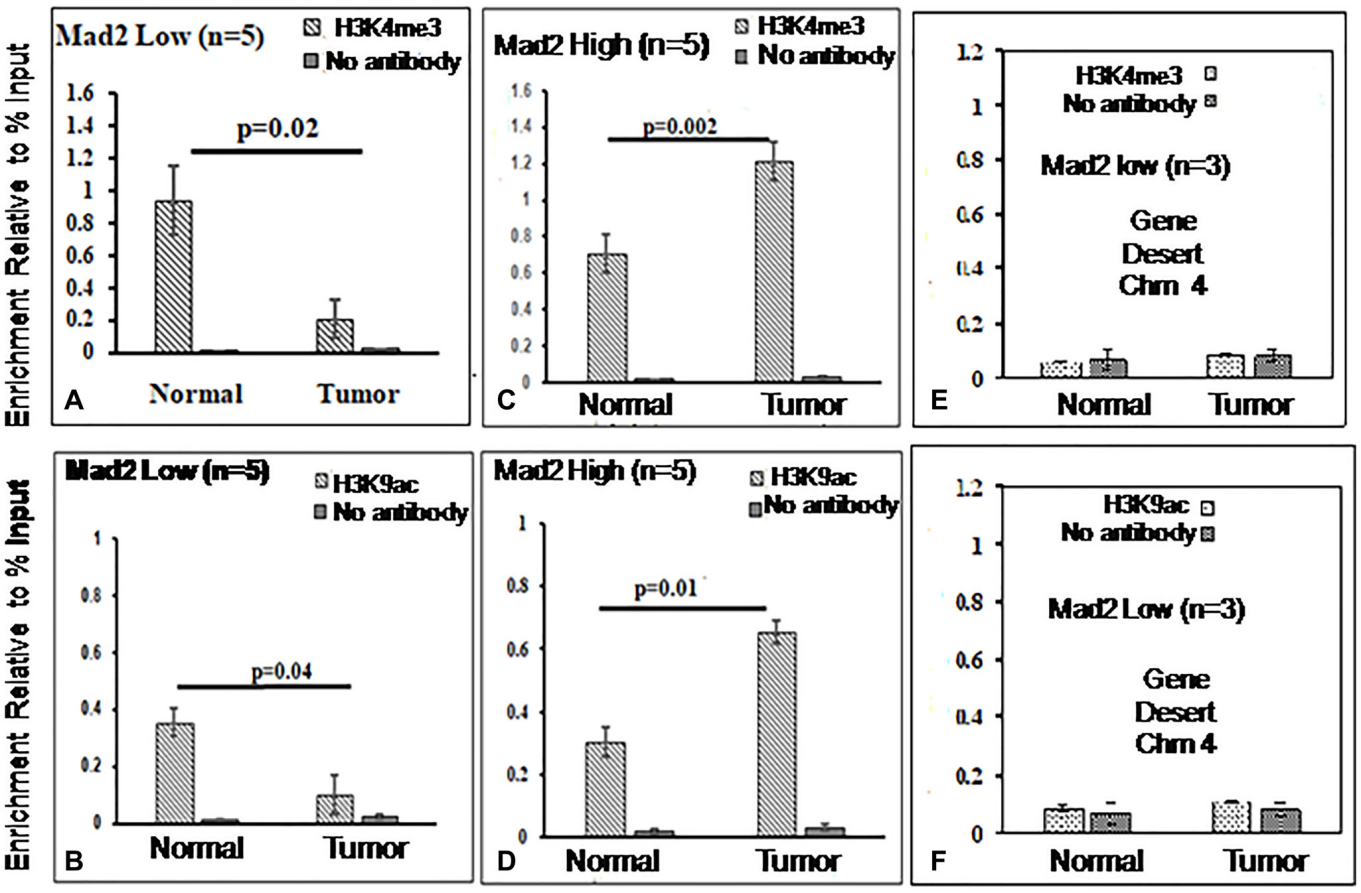
ChIP analysis of histone H3 methylation and acetylation at the core promoter region of the Mad2 gene. (**A**, **B**) ChIP-qRT-PCR assays for H3K4me3 and H3K9ac, in Mad2 low expression group of cancer samples and (**C**, **D**) for Mad2 high expression group of cancer samples. Chromatin was cross-linked, fragmented and immunoprecipitated with no antibody (as a negative control), with Histone 3 antibody (as a positive control) or anti-H3K4me3 and H3K9ac ChIP-grade antibodies. The purifed DNA was used to amplify with primer pairs covering the core promoter region (−57 to −190) of the Mad2 promoter by qPCR. As input, 10% diluted chromatin fragments were retained and used in qPCR for the enrichment analysis. The percentage of input values represents the mean of n number of cancer samples ± SEM. Data were analyzed using one-way ANOVA with Tukey’s multiple comparison pos*t*-tests. *P* values less than 0.05 are considered significant. (**E**, **F**) ChIP analysis of histone H3K4 methylation and H3K9 acetylation at the gene desert region of human chromosome 4 in esophageal cancer cells. ChIP-qRT-PCR assays for H3K4me3 and H3K9ac recruitment in the gene desert regions were analysed and served as negative control.

**Figure 5:**
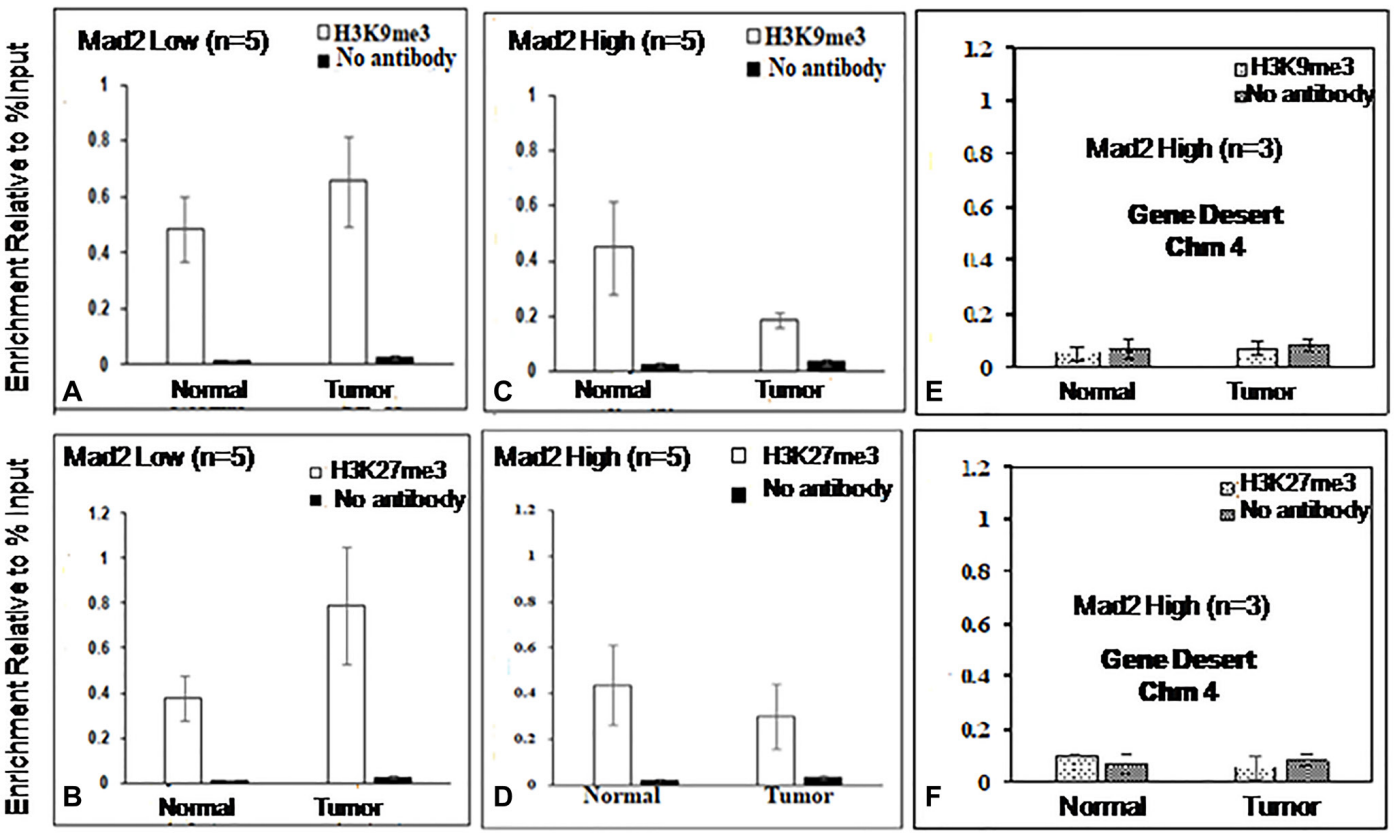
ChIP analysis of histone H3, 9 and 27 methylation at the core promoter region of the Mad2 gene. (**A**, **B)** ChIP-qRT-PCR assays for H3K9me3 and H3K27me3, in Mad2 low expression group of cancer samples and (**C**, **D**) for Mad2 high expression group of cancer samples. Chromatin was cross-linked, fragmented and immunoprecipitated with no antibody (as a negative control), with Histone 3 antibody (as a positive control) or anti-H3K9me3 and anti-H3K27me3 ChIP-grade antibodies. The purified DNA was used to amplify with primer pairs covering the core promoter region (−57 to −190) of the Mad2 promoter by qPCR. As input, 10% diluted chromatin fragments were retained and used in qPCR for the enrichment analysis. The percentage of input values represents the mean of n number of cancer samples ± SEM. Data were analyzed using one-way ANOVA with Tukey’s multiple comparison pos*t*-tests. *P* values less than 0.05 are considered significant. (**E**, **F**) ChIP analysis of histone H3K9 and 27 tri- methylation at the gene desert region of human chromosome 4 in esophageal cancer cells. ChIP-qRT-PCR assays for H3K9me3 and H3K27me3 recruitment in the gene desert regions were analysed and served as negative control.

The assays were analyzed using qPCR, specifically targeting the core promoter region (−57 to −190 bp) of the Mad2 gene. Figures 4 and 5 display the outcomes of ChIP experiments, indicating the enrichment of histone marks on the Mad2 promoter in terms of % input. ChIP experiments reveal a significant decrease in H3K4me3 and H3K9ac and an increase in H3K9me3 and H3K27me3 levels in tumor tissue compared to adjacent normal tissue where Mad2 was underexpressed (Figure 4A, 4B; Figure 5A, 5B).

In contrast, in the samples where Mad2 was overexpressed, the levels of H3K4me3, and H3K9ac were increased significantly and the levels of H3K9me3 and H3K27me3 were decreased (Figure 4C, 4D; Figure 5C, 5D).

**Figure 6:**
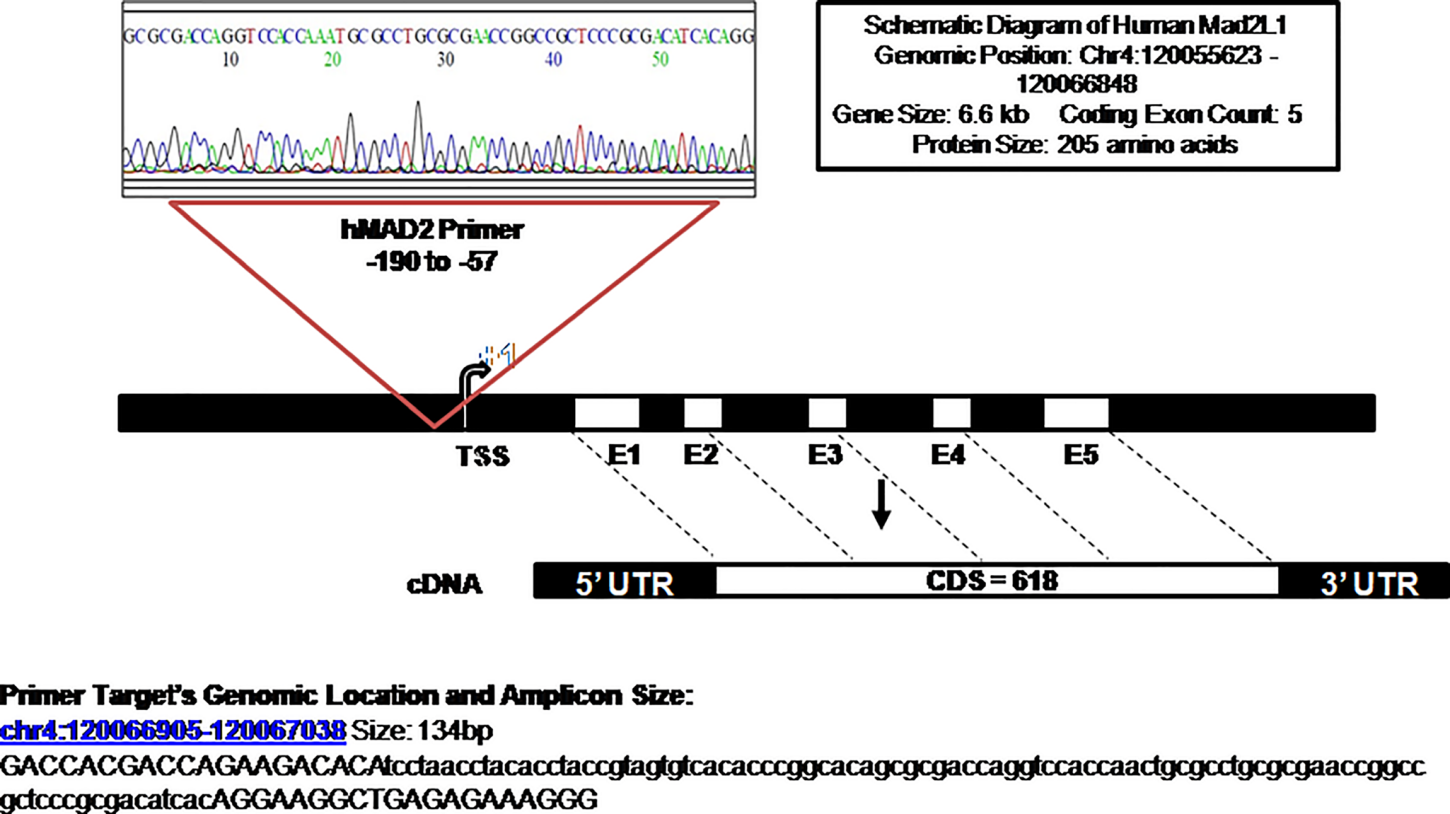
Sequencing of qRT-PCR products: schematic diagram depicting the position of the qRT-PCR product that was sequenced and matched in the promoter region (−57 to −190) amplified by the primer set of the human Mad2 gene. The transcription initiation site (+1) is indicated with an arrow and 5 exons are also shown. The sequence details of the amplified product are shown below.

DNA fragments recovered from immunoprecipitated samples for H3K4me3, H3K9ac, H3K9me3 and H3K27me3 show significant epigenetic modifications in the promoter core region. ChIP-assay in chromosome 4’s desert region as a negative control revealed negligible DNA fragments in tumor and normal samples (Figure 4E, 4F; Figure 5E, 5F). The Histone3 antibody was used as a positive control, and the DNA fragments retrieved from immunoprecipitated samples were similar in both normal and tumor cells (Supplementary Figure 1A, 1B). The qPCR product, 134 bp, was sequenced and blasted (NCBI nucleotide blast) with human genomic sequences, matching the human Mad2 gene promoter region on chromosome 4q27 (Figure 6).

## DISCUSSION

The present study found that 41% of esophageal cancer samples overexpressed Mad2, while 50% showed downregulation out of total 32 samples that were analyzed. In the past, the expression of MAD2 in 49 oral squamous cell carcinoma cases was analyzed using immunohistochemistry, and findings were compared with clinicopathological parameters and DNA ploidy. It was observed that overexpression of Mad2 in 37% of cases was linked to a more malignant phenotype, despite equal aneuploidy induction in both overexpressed and underexpressed samples [21]. This study also demonstrated equal aneuploidy induction regardless of Mad2 expression status, but aimed to identify the mechanism causing altered Mad2 expression during carcinogenesis.

Interestingly, three patients in the current cohort displayed similar levels of chromosomal instability (CIN) without altering Mad2 gene expression between normal and tumor cells. Exposure to raw AN and lime for 240 days or more can cause CIN and stomach cancer in mice with securin upregulation [11]. Securin upregulation in non-target cells like PBLs, can also cause CIN in humans, with the habit of chewing AN and lime with or without tobacco [12, 13] has also been reported. Therefore, securin overexpression can compromise CIN even if the Mad2 gene does not show any change in the expression pattern.

In this study, PBL cells were fixed at 88 h because a higher frequency of PAS and aneuploidy was observed in 88 h fixation samples than in 56 h. This indicates that cell division is required to acquire such an abnormal karyotype [12]. A similar phenomenon is observed in many malignancies, including oral and esophageal squamous cell carcinoma [22, 23]. Thus, impaired SAC genes and chromosome segregation machinery disruptions may initiate carcinogenesis, which can potentially be detected in non-target cells like human PBLs and mouse bone marrow cells [10–12].

Mitotic checkpoint impairment is linked to tumorigenesis, but MAD2 gene mutations or deletions are rare in human cancers [24], except gastric cancer [25]. Therefore, the regulation of Mad2 gene transcription in human cancer cells differs from normal cells, emphasizing the need for proper control for normal growth [26]. Previous studies suggest various mechanisms for Mad2 gene deregulation, including genomic rearrangements, altered gene dosage, promoter methylation, and transcriptional defects in Mad2 production [17, 18].

The trigger for Mad2 downregulation in cancer samples is unclear, as earlier studies showed it occurs during hypoxia and not due to promoter hypermethylation [27]. Further studies found no evidence of Mad2 promoter methylation in breast and prostate cancer cells or in clinical materials [27]. Meanwhile, it was demonstrated that mice with a single Mad2 allele are viable with chromosomal abnormalities and prone to tumor growth [7, 28]. Therefore, in order to study the changes in the genome accompanying Mad2 deletion, we performed LOH analysis and noted around 40% deletion of the microsatellite marker D4S2975 located in 4q27 where the human gene Mad2 has been assigned [29]. Hammoud et al. (1996) [30] found LOH frequency in esophageal adenocarcinoma at 54.5–65% in 4q21-35 region. In hepatocellular carcinoma, LOH frequency was scored at 33% [31] and 50% [32] in 4q26-27 region. These results strongly indicated that more than one putative tumor suppressor genes may be located at 4q. Some studies have implicated the tumor suppressor human Cyclin A gene (CCNA) in this region of 4q [33], and since the function of Mad2 aligns with tumor suppressor definition, this locus could conceivably be involved in lesions in cancer patients that map to 4q27. It was demonstrated that genetic manipulation of Mad2 function partially leads to premature degradation of Securin and separation of sister chromatids, resulting in aneuploidy [7]. The present results show premature anaphase and aneuploidy in underexpressed Mad2 samples, suggesting 4q27 region involvement in esophageal cancer samples.

Mad2 overexpression is considered to be a more common event in human cancers, causing aneuploidy and tetraploidy [5], and associated with poor prognosis due to its E2F target gene expression in tumors lacking Rb activity [17, 34]. Several SAC genes, including Mad2, are E2F targets and 40% of the present tumor samples showed Mad2 overexpression along with higher Rb-phosphorylation and E2F expression. Mad2 overexpression is observed after adenovirus E1A-mediated inactivation of pRb, leading to stimulation of E2F-dependent transcription of Mad2 mRNA [17]. It was reported that altered expression of Rb and E2F is associated with tobacco/betal quid use as well as aggressive oral cancers [35]. It was revealed that exposure to raw AN with lime can create a relaxed chromatin structure, potentially enhancing transcription of key genes linked to carcinogenesis [36, 37]. Therefore, studies on histone covalent modification patterns are crucial for understanding gene promoter activity and cancer links [38]. A study found that exposure to raw AN and lime can upregulate the Securin gene, a potential E2F1 target, in mice’s stomach tissue through posttranslational histone H3 modifications in securin gene promoter regions [13].

The present ChIP-qPCR data indicate a significant increase of H3K4 trimethylation and H3K9 acetylation in overexpressed samples of Mad2 gene’s core promoter region. Interestingly, both these posttranslational modifications of histones were reduced where Mad2 expression was underregulated. Similarly, both the repressor histone such as H3K9me3 and H3K27me3, were reduced where Mad2 was overexpressed and increased where Mad2 was underexpressed. It is important to mention that several attempts were made earlier to unravel the mechanisms of Mad2 gene deregulation, and this is the first time we have analyzed the histone 3 covalent modification patterns in the core promoter region of the Mad2 gene in esophageal cancer samples. The study reveals that H3K4 trimethylation in active promoters is linked to transcriptional activation, epigenetic modification, and oncogenesis [39]. Misregulated HATs or HDACs change acetylation signalling and cause chromatin decompaction, abnormal gene expression, and DNA damage responses [40, 41]. Repressive histones H3K9me3 and H3K27me3 impede transcriptional elongation and gene expression [42]. The study reveals that a decrease in H3K9me3 leads to an increase in H3K9ac, indicating that the Mad2 promoter’s transcriptionally active state is influenced by H3K9 methylation and acetylation in response to AN-mediated stimulation. Moreover, transcription facilitator H3K9 acetylation is mutually exclusive to transcriptionally repressive H3K9 methylation [43]. The data show a unique pattern of histone 3 covalent modification in Mad2 expression deregulation. The study reveals a distinct pattern of histone 3 covalent modification in Mad2 expression deregulation, indicating that transcription facilitator H3K9 acetylation is exclusive to transcriptionally repressive H3K9 methylation [43].

This study did not analyze the expression patterns of different enzymes that are involved in posttranslational modifications of histones since previous studies on similar samples in this laboratory showed higher expression of KMT2A and KAT2A and decreased HDAC3 levels after raw AN exposure [13]. Another study revealed increased expression of enzymes regulating histone methylation and acetylation in esophageal cancer patients with AN chewing habits [44]. Therefore, it seems that all these enzymes, like KAT2A, KMT2A, p300, and P300/CBP- associating factor (PCAF), will be enhanced in the present sample irrespective of Mad2 expression. It is also worth mentioning that raw AN exposure hyperphosphorylates pRb, enabling E2F1 transcription of target genes like PTTG1/securin and Mad2 but it is not clear about the role of hyperphosphotylated pRb in Mad2 underexpressed samples. Over 300 Rb1 interactors are involved in multiple signalling complexes [45], inhibiting E2F transcriptional activity by blocking transcription factors, histone acetylases [46, 47], and recruiting histone-modifying and chromatin-remodelling factors to promoters [48]. Further research is needed to understand the role of pRb in Mad2 downregulation, as it prevents chromatin decompaction at the Mad2 gene promoter region.

The present study observed that Mad2 expression levels, regardless of high or low, can serve as a clinically useful biomarker for identifying patients with chromosomal abnormalities. The data indicate that the disruption of 4q27 where the Mad2 gene is located, is a crucial genetic event for reducing Mad2 expression in raw AN-induced esophageal carcinogenesis. On the other hand, raw AN exposure mediated Mad2 overexpression might be due to deregulation of the Rb-E2F1 circuit, resulting in the elevation of H3K4me3 and H3K9ac in the Mad2 gene promoter region. It has been demonstrated that low Mad2 levels are linked to cisplatin resistance, while high levels indicate drug sensitivity [49, 50]. Patients with overexpressing MAD2 may benefit from direct MAD2 targeting, restoring apoptotic signaling [51]. Therefore, depending on Mad2 expression levels, patients can be divided into different treatment categories to maximize treatment outcomes. Further research is needed to understand the cellular processes altered by RB1 mutations that suppress Mad2 expression.

## MATERIALS AND METHODS

### Patients and tissue samples

Esophageal squamous cell carcinoma (ESCC) biopsies (*n* = 131) and peripheral blood samples were collected from patients at Nazareth hospital in Shillong, India, after their consent and individual interviews. The detail information on patients from whom the samples were collected is given in the Supplementary Section (Supplementary Table 1). Out of 131 samples, 99 were used for LOH analysis at 4q25-28 with six microsatellite markers. The 99 samples were categorized into 33 raw AN chewers and 66 raw AN chewers with tobacco users, who either smoke or chew tobacco.

The other 32 cancer biopsies and adjacent normal tissues were used for the expression analysis by qRT-PCR, immunohistochemistry (IHC), and ChIP-qPCR analyses and their blood samples were used for metaphase preparation. Before taking the biopsies, the consent for their participation were received. Some of these collected samples were from patients with occasional drinking habits and histologically all the samples were identified as squamous cell carcinoma.

### Lymphocyte culture procedure and preparations of metaphases

Peripheral blood was collected from 42 donors of which 10 were noncancerous and nonchewers and 32 were from esophageal cancer patients. Heparinized blood was mixed with Rosewell Park Memorial Institute 1640 medium (Biological Industries Ltd., Israel), L-glutamine (Sigma, USA), and 10% heat inactivated foetal calf serum (Biological Industries Ltd., Israel), were stimulated with phytohaemagglutinine (Gibco, Life Technologies) and incubated at 37°C for 88 hours. For each patient, two lymphocyte cultures were prepared.

Colcemid was added (0.01 μg/ml) to cultures during the last 3h, and cells were harvested using a conventional method. Briefly, cells were treated with KCl (0.56%; prewarmed at 37°C), fixed in acetic acid and methanol (1:3), air-dried, stained with 5% Giemsa, air-dried, and mounted on synthetic medium.

### Quantitative real-time PCR

Total RNA was isolated from tumour and normal tissue samples with Trizol, purified using RNeasy Mini Kit (Qiagen GmbH, Hilden, Germany), and cDNA synthesis was performed using QuantiTect Reverse Transcription kit (Qiagen GmbH, Hilden, Germany). The qRT-PCR was conducted using a Bio-Rad CFX96 Real-Time PCR Detection System using SYBR Green PCR Master Mix (Thermo Fischer Scientific, Massachusetts, USA). This analysis utilized Mad2 primers and GAPDH as reference genes, with primer sequences listed in Supplementary Table 2 (Supplementary Information) for further details.

### Immunohistochemistry (IHC)

IHC study was carried out to validate qRT-PCR observation of Mad2 gene expression using nine samples from over- (*n* = 4) and under (*n* = 5) expression groups. Pathologists and Head and Neck Surgery Department at Nazareth Hospital reviewed esophageal cancer samples and control tissue to confirm diagnoses and select representative blocks for immunohistochemical analyses. The study involved dehydrating, paraffin embedding, sectioning tumour and normal tissue with a microtome (Leica), incubating sections with anti-Mad2 (SC-374131; Sant Cruz Biotechnology, USA), anti-Rb phosphorylation (SC-271930; Sant Cruz Biotechnology, USA), and anti-E2F1 (SC-22820; Santa Cruz Biotechnology, USA) primary antibodies, and performing IHC analysis with a Strept-Avidin Biotin Kit (Dako) (see Supplementary Information).

### DNA isolation and analysis of LOH

Genomic DNA was extracted from 99 tumour samples and lymphocytes (isolated with HISTOPAQUE– 1077; Sigma, USA) from peripheral blood samples using 1X NET Buffer (10 mM Tris (pH 8.0)); 25 mM EDTA [100 mM NaCl (pH 8.0)]; 10% (w/v) SDS (Sigma, USA); Proteinase K ((20 mg/ml) (Bangalore Genei, India)), and the standard extraction procedures [52]. Six dinucelotide polymorphic markers were selected for 4q25-28 region of the chromosome based on map position and heterozygosity (Gene Map 99). Each microsatellite primer was obtained from Sigma, USA, with sequences provided in Supplementary Table 3 (Supplementary Information). The PCR reaction involved a 10 μl volume with MgCl2 (1.5–2.5 mM), primers (4 pmol of each primer; one-fifth of one of which was end-labelled with [γ^32^P]dATP), dNTP (0.2 mM), DNA (25 ng), and AmpliTaq DNA polymerase (0.3 units; Perkin-Elmer Corp., Branchburg, NJ), amplification for 30 cycles at varying temperatures (ranging from 52 to 60°C).

The PCR products were denatured in formamide containing sequence stop buffer, electrophoresed (6% urea containing polyacrylamide gel), and dried the gels for autoradio-graphed (4–16 h). LOH was scored after considering a 50% reduction in signal intensity of one allele in the tumor compared to constitutional alleles in the blood (by visual analysis and also by using Kodak GelLogic Imaging software).

### Chromatin immunoprecipitation assay (ChIP)

ChIP assays were conducted on human esophageal cancer samples and normal tissues to detect posttranslational histone modification patterns in the Mad2 gene’s core promoter region. Esophageal cancerous samples with both Mad2 overexpression (*n* = 5) and underexpression (*n* = 5) were lysed, sonicated, and incubated overnight at 4°C with antibodies specific to H3K4me3 (ab8580, Abcam, UK), H3K9Ac (ab12179), H3K9me3 (ab8898), H3K18Ac (ab1191) and Histone 3 (ab1791) with protein A/G beads (Pierce™ Protein A/G Agarose, Cat no. 20421). The methodology of ChIP is thoroughly explained in the Supplementary Section.

The purified DNA from immunoprecipitated samples underwent SYBR green RT-qPCR (BioRad CFX system) using primers ranging from −57 bp to −190 bp to identify the Mad2 promoter region as confirmed by sequencing (Science genome browser) of the qPCR products:

(Forward Primer: 5′-GACCACGACCAGAAGACACA-3′; Reverse Primer: 5′-CCCTTTCTCTCAGCCTTCCT-3′).

RT-qPCR was also performed on immunoprecipitated ChIP samples, using a negative control set of primer sets representing the desert regions of chromosome 4 in human samples. The primer sets were designed from the downstream region of the PCDH7 gene (location 30.7 to 31.1Mb) where 5.2 Mb is the gene desert region of chromosome 4 [53] is present. The primers sequences are (F: 5′-CATCACGCCCGGCTAATTTT-3′; R: 5′-TCATGCCTGTAATCCCAGCA-3′) and the product size was 132.

### Scoring and statistical analysis

Over 100 metaphases were studied (except one case), with chromosome counts performed on each sample, with values expressed as mean ± SD or mean ± SEMs.

The study used unpaired Student’s *t*-test with GraphPad Prism software 5.1. to analyze differences between control and treated groups, with significance determined by a *P* value of 0.05 or less.

The study analyzed gene expression in normal and cancerous cells using paired Student’s *t*-test. One-way ANOVA was used in determining the significance of Histone3 K4-trimethylation, H3K9-acetylation, and H3K27-trimethylation levels between the normal and cancerous groups. The Tukey test was utilized for post hoc analysis, with results displayed as means ± SEM, and statistical significance was determined at *P* < 0.05.

## SUPPLEMENTARY MATERIALS


