# Supplementary material for: Differential expression of Mad2 gene is consequential to the patterns of histone H3 post-translational modifications in its promoter region in human esophageal cancer samples
Source: Oncotarget. 2024 Feb 5;15:76–89. doi: 10.18632/oncotarget.28554 (PMC10852063; doi:10.18632/oncotarget.28554)
Supplement: Supplementary file 1 [file oncotarget-15-28554-s001.pdf]

Differential expression of Mad2 gene is consequential to the patterns of histone H3 post-translational modifications in its promoter region in human esophageal cancer samples

SUPPLEMENTARY MATERIALS

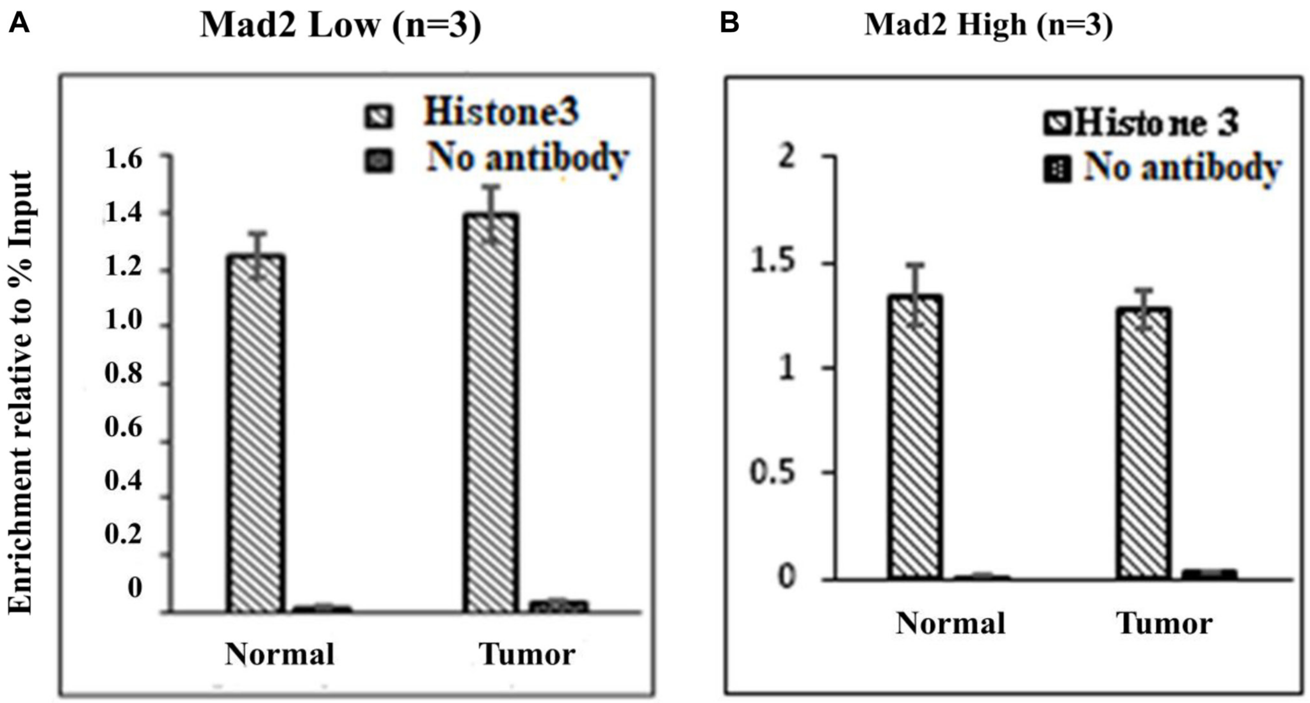

Supplementary Figure 1: ChIP-qRT-PCR assays with Histone 3 antibody as a positive control in the core promoter region of the Mad2 gene in 3 samples each of the Mad2 low (**A**) and Mad2 high (**B**) expression groups of esophageal cancer samples.

SUPPLEMENTARY INFORMATION

Experimental procedure

Patients and tissue samples

The detail information on patients from whom the samples were collected is given in the Supplementary Table 1.

Quantitative real-time PCR

The primers of target genes used for this analysis were Mad2, and GAPDH (as the reference gene). The primer sequences are listed in Supplementary Table 2.

Immunohistochemistry (IHC)

Formalin fixed, paraffin embedded tissues were sectioned (5 µm thickness) for IHC staining. Slides were deparaffinized, rehydrated and treated with PBS at 4°C for 5 min, with 3% hydrogen peroxide added to inhibit intrinsic peroxidase activities. Antigen retrieval was done with 0.01% sodium-citrate buffer followed by blocking in PBST containing 0.1% BSA and 10% FBS. The samples were reacted with primary antibody (1:100) for overnight and rinsed with PBS. Slides were incubated with appropriate biotinylated secondary antibody for 1hr at room temperature. Following washing, slides were treated with streptavidin-HRP (1:1000) and subsequently washed (PBS containing 0.1% Tween-20) and color was developed with DAB+H_2O_2. Slides were counterstained with haematoxylin, washed and mounted in DPX (Sigma-Aldrich, USA).

Two investigators assessed staining intensity in histologic sections, categorizing chromogenic immunolabeling into four groups: 0 (no labelling), 1+ (weak labelling), 2+ (moderate labelling), and 3+ (strong staining; observable with 10× objective). Semi-quantitative staining analysis was done by H-score by counting 500 cells from ten different fields in the slides considering low, moderate and higher intensity of Mad2, pRb-phosphorylation and E2F1 expression. The percentage of positive cells with a given intensity for each sample was determined independently by a pathologist and a trained reader. A single manual H-score based on a scale of 0 to 180 was generated for each labelled section by taking the sum of the percentage of cells labelling 1+, double the percentage of cells labelling 2+, and triple the percentage of cells labelling 3+ (H-Score = ((%3+) × 3) + ((%2+) × 2) + (%1+)).

Analysis of LOH

The sequences of each microsatellite primer for 4q are given in the Supplementary Table 3.

Quantitative Real-time PCR

cDNA systhesis was performed from 1 µg of total RNA from each sample using Quantiscript Reverse Transcriptase, Quantiscript RT-buffer and RT Primer-mix of the QuantiTect Reverse Transcription kit (Qiagen GmbH, Hilden, Germany) according to the manufacturer's protocol. Quantitative real-time PCR was performed on 96-well optical reaction plates (Applied Biosystems, Darmstadt, Germany) using a StepOnePlus amplification and detection system (Applied Biosystems). The real-time RT-qPCR reactions were performed with reagents containing SYBR green and primer sets and the following conditions were used: 95°C for 5min, 40 cycles of 95°C for 30 s, 60°C for 30 s and 72°C for 30 s. The gene copy numbers of Mad2 were calculated using a standard curve that was constructed using the OE33 cell line (obtained from European Collection of Authenticated Cell Cultures, Cat No. 96070808; maintained in RPMI 1640 medium with 2 mM Glutamine and 10% Foetal calf serum). The 2−ΔΔCT method was used as a relative quantification strategy for qPCR data analysis.

Chromatin immunoprecipitation and ChIP-qRT-PCR

Chromatin immunoprecipitation (ChIP) assays were performed as described earlier (13). Scraped and minced cells from human esophageal cancer samples and adjacent normal tissues (12 mg for each ChIP reaction) were exposed to 1% formaldehyde for 20 min, followed by homogenized (15–20 strokes with Dounce homogenizer) in homogenization Buffer (10 Mm HEPES at Ph 7.9, 0.5% NP-40, 1.5 Mm MgCl2, 10 Mm KCl, 0.5 Mm DTT, 1 Mm PMSF and 1X PIC (Protease inhibitor cocktail- 1 Mm PMSF, 1 µg/ml aprotinin and 1 µg/ml pepstatin A) on ice (1 ml per 200 mg tissue). After centrifugation at ~960 g for 5 min at 4°C, supernatant was removed. Cell pellets were lysed in nuclei lysis buffer (50 Mm Tris-HCl (Ph 8.0), 10 Mm EDTA, 1% EDTA, 1X PMSF and 1X PIC (protease inhibitor cocktail-1 Mm PMSF, 1 µg/ml aprotinin and 1 µg/ml pepstatin A; Sigma P8340; USA) and sonicated (Sonicator UP100h Hielscher, 15–20 cycles, 30 Sec ON and 60 Sec OFF. Amplitude:60%) with Shearing buffer (50 Mm Tris-HCl (Ph 8.0), 10 Mm EDTA, 1% EDTA, 1X PMSF and 1X PIC) on ice (100 Ml/20 mg tissue). After centrifugation at ~21,000 g for 10 min at 4°C, supernatant was collected. The supernatant was diluted with an equal volume of ChIP dilution buffer (1.1% Triton X-100, 1.2 Mm EDTA, 16.7 Mm tris-HCl (Ph 8.0), 167 Mm NaCl, PMSF and PIC) and distributed equally for each antibody. 10% of diluted chromatin kept as Input. Immunoprecipitation was performed with antibodies specific to H3K4me3 (ab8580), H3K9ac (ab12179),

Supplementary Table 1: Details about the esophageal cancer patients used in this study

Characteristics	Number	
**Esophageal cancer**	**99 32 = 131**	
Male/Female	59/40	20/12
Age Mean ± SD	**51.79 ± 10.24**	
**Habits**		
Raw AN chewing	33	10
Raw AN + Tobacco	66	22
**Histopathology**		
Well differentiated	82	25
Moderately differentiated	17	7

Supplementary Table 2: The primer sequences for detecting the candidate genes encoding human Mad2 and GAPDH.

Target name	Primer sequence	Product size
Mad2L1	F: 5′-GGTCCTGGAAAGATGGCAG-3′<br>R: 5′-ATCACTGAACGGATTTCATCC-3′	111 bp
GAPDH	F: 5′-ACAGTCCATGCCATCACTGCC-3′<br>R: 5′-GCCTGCTTCACCACCTTCTTG-3′	266 bp

Supplementary Table 3: The primer sequences of microsatellite markers of chromosome 4q

Microsatellite markers	Primer sequences	PCR product size (bp)
**D4S407**	F: 5′-ATAATATCCTTTGATCCTTTCGCTA-3′<br>R: 5′-AAATTTGGTTATTTTTAAGCAAACT-3′	111–135
**D4S1612**	F: 5′-AAGGCTTTATTCNCTTATTGTT-3′<br>R: 5′-GGTCCAAAGACAGGTCAAA-3′	160–186
**D4S1522**	F: 5′-CCGAATCTCAAAAAATGTTAG-3′<br>R: 5′- AATGCCATAAAATCAAGTAAAG-3′	180
**D4S2975**	F: 5′-CTGGGCTCAAGTGATTTTC-3′<br>R: 5′-CAGCTGGGACAATGGTGT-3′	226–276
**D4S1615**	F: 5′-CCTTGGGTCAGCCACATATC-3′<br>R: 5′-CACTCAGAACAGAAACTTGGGT-3′	115–125
**D4S424**	F: 5′-GCGCTCTTGGTATATGGTACAG-3′<br>R: 5′-TGTGGGCAACGTCACTC-3′	178–192

H3K9me3 (ab8898), H3K18ac(ab1191) and Histone 3 (ab1791) with protein A/G beads (Pierce™ Protein A/G Agarose, Cat no. 20421) incubated for 7–8 hours at 4°C on orbital rotor while only protein A/G beads with cell lysate without antibody kept as control in the same process. After incubation, precipitates were sequentially washed with Low Salt Buffer (150 Mm NaCl, 0.1% SDS, 1% Triton X-100, 2 Mm EDTA, 20 Mm Tris-HCl (Ph 8.0)), High Salt Buffer (500 Mm NaCl, 0.1% SDS, 1% Triton X-100, 2 Mm EDTA, 20 Mm Tris-HCl (Ph 8.0)), LiCl buffer (0.25 M LiCl, 1% NP-40, 1% sodium deoxycholate, 1% Mm EDTA, 10 Mm Tris-HCl (Ph 8.0)) and 1X TE (10 Mm Tris-HCl, Ph 8.0, 1 Mm EDTA). DNA was then eluted with 1% SDS and 0.1 M NaHCO3. The eluates together with 5M NaCl incubated for at least 6 hours to overnight at 65°C to reverse the formaldehyde cross-linking. Next day, RNase (1 mg/ml) added to eluates incubated at 37°C for half an hour followed by 1 M Tris-Cl, 0.5 M EDTA and Proteinase k (20 mg/ml) incubation at 45°C for 2 hours. Chloroform in the equal volume added to the above contents to remove remaining proteins and further ethanol and salt purification of the aqueous phase was

done to precipitate the pure DNA. DNA fragments were then dissolved in 1X TE and stored at −20°C. Purified DNA was analysed using Quantitative Real Time PCR using reagents containing SYBR green. ΔΔCt method (BioRad CFX system) using primer for human Mad2 gene promoter designed from UCSC browser and Primer 3 software (Version 0.4.0). Samples were heated to 95°C for 5 min and then amplified for 45 cycles at 95°C for 30 s, 60°C for 30 s and 72°C for 30 s. Immunoprecipitated DNA was detected by qPCR and normalized to input DNA. Enrichment was calculated relative to input. qPCR products were purified using a SIGMA PCR clean up kit (NA 1020) and submitted for sequencing to Agrigenome, Kochi, India.
